# Effect of K_2_ZrF_6_ Concentration on the Two-Step PEO Coating Prepared on AZ91 Mg Alloy in Alkaline Silicate Solution

**DOI:** 10.3390/ma13030499

**Published:** 2020-01-21

**Authors:** Zeeshan Ur Rehman, Bon Heun Koo, Yeon-Gil Jung, Je Hyun Lee, Dongjin Choi

**Affiliations:** 1 School of Materials Science and Engineering, Hongik University, Sejong-si 30016, Korea; Zeeshan.physics@gmail.com; 2 School of Material Science and Engineering, Changwon National University, Changwon 642968, Korea

**Keywords:** PEO, magnesium, K_2_ZrF_6_, corrosion, hardness, two-step

## Abstract

In this study, a two-step Plasma Electrolytic Oxidation (PEO) method with constant primary step parameters and varying K_2_ZrF_6_ concentration in the secondary electrolyte solution was carried out to obtain a hard and dense coating on AZ91D alloy. For lower concentrations of K_2_ZrF_6_, a mixture of granular and pancake structure with higher surface porosity was obtained. Increasing the concentration up to 6 g/L caused the granular structure to disappear and a significant decrease was observed in the surface porosity as well as inner layer damage. Due to the compact inner layer structure, significant improvement in the corrosion resistance and mechanical properties of the AZ91 was observed with K_2_ZrF_6_ concentration. Highest values of hardness and corrosion resistance, i.e., 1589.45 HV and 386.30 × 10^3^ Ω cm^2^, respectively, were obtained for a 6 g/L K_2_ZrF_6_ concentration.

## 1. Introduction

PEO (Plasma electrolytic oxidation) process is an electrochemical coating process involving a number of reactions such as; electro-, thermal-, and plasma-based chemical reactions. Due to the involvement of these high-temperature processes, PEO coatings are much better than conventional anodic films in various aspects such as thickness, hardness, interface adhesion, wear and corrosion protection, etc. In addition, the PEO process is considered very efficient for its short manufacturing time, eco-friendly nature, excellent throwing power, cost-effective properties and flexibility to deposit coating layers on both regular and irregularly shaped substrates [1,2,3]. However, due to micro-arc discharges, gas liberation and thermal processes during the PEO process, the coatings usually displayed large defects, cracks and pores [4]. These defects are generally detrimental to all the useful applications of PEO coatings; however, in particular, they are harmful for the anti-corrosion property of the coatings. Researchers discovered that PEO-coated metals experience constant corrosion degradation during long-term immersion in chloride solution and reasoned that the penetration of the corrosive solution through the porous microstructure of the coatings was a major cause [5]. Therefore, extensive studies have been carried out in multiple directions to optimize the PEO coatings for enhanced anti-corrosion properties, e.g., coating composition and microstructure [6,7], barrier layer property [5,8], coating thickness, etc. Thus, new electric parameters [9,10], electrolyte compositions [7,11,12] and pre-treatments [13,14] have been developed to enhance the corrosion resistance of PEO coatings. Recently, the use of K_2_ZrF_6_ additive and two-step PEO coating have achieved prominent interest among the PEO research community. Yang et al. deposited PEO coatings on titanium alloy and found that K_2_ZrF_6_ additives showed a self-healing microstructure and enhanced mechanical properties [15]. Comparing various Zr- based additives, Haihe et al., observed that K_2_ZrF_6_ produced better corrosion properties than ZrOCl_2_ and Zr(NO_3_)_4_ due to formation of a denser internal film and a more uniform surface [16]. The anti-corrosion properties of K_2_ZrF_6_ were attributed to the activating surface of the substrate by the K_2_ZrF_6_ additives. Serval other authors have previously carried out experiments using K_2_ZrF_6_ in order to understand the properties and significance of the K_2_ZrF_6_ additives in primary step coatings [17,18,19,20,21,22,23].

Similarly, in terms of the two-step PEO coating, several authors demonstrated significant achievements. For instance, Kang et al., reported microstructural and phase profile characteristics of the two-step coating using acidic K_2_ZrF_6_ electrolyte solution [24]. Tsunekawa et al. used acidic K_2_ZrF_6_ solution and discussed the mechanical properties of PEO coatings on titanium alloy [25]. Feryar et al. treated AZ31 magnesium alloy in acidic K_2_ZrF_6_-based electrolyte solution and obtained enhanced anti-corrosion properties [26]. These reports offer a motivation toward the further investigation of K_2_ZrF_6_-based two-step PEO coating in various directions, such as combination of K_2_ZrF_6_ with other additives, K_2_ZrF_6_-based PEO coatings in alkaline solution, the effect of K_2_ZrF_6_ concentration on the PEO coatings. Consequently, in this work, the effect of K_2_ZrF_6_ concentration on the two-step PEO coatings on AZ91 magnesium alloy in alkaline solution has been investigated. 

## 2. Experimental Details

### 2.1. Specimens Pre-Treatment and Electrolytes

Coupons of AZ91D alloy (Al 9.1 wt.%, Zn 0.85 wt.%, Mn 0.27 wt.%, Fe ≤ 0.02 wt.%, others ≤ 0.01 wt.%, Mg balance) with a working area ~9.5 cm^2^ were used. The pretreatment of the coupons includes; Polishing with SiC paper (up to grade 2000), washing in deionized water, decreasing in ethanol. Two different electrolytes were used in the experiment, named Bath-1 and Bath-2, respectively. Bath-1 was composed of N_2_SiO_3_·10H_2_O 12 g/L, NaOH 3.5 g/L, Na_2_SiF_6_ 0.3 g/L, and Bath-2 was composed of K_2_ZrF_6_ (2~8 g/L, Na_2_SiO_3_·10H_2_O 12 g/L, NaOH 3.5 g/L, Na_2_SiF_6_ 0.3 g/L.

### 2.2. PEO Coating Deposition by Primary and Secondary Step

**Figure 1:**
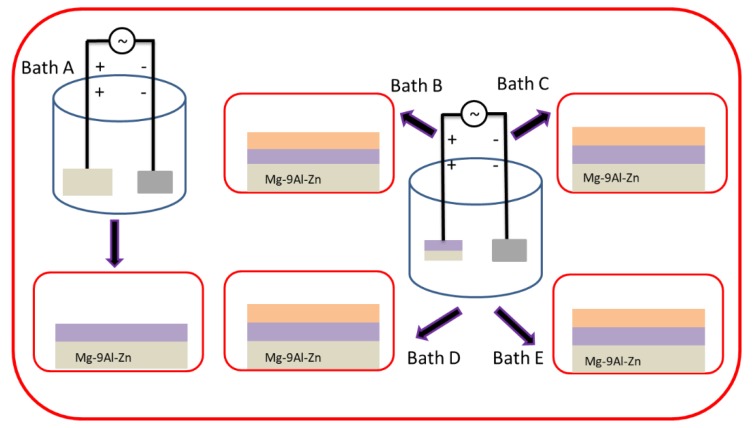
Schematic illustration of PEO process with various K_2_ZrF_6_ concentration.

To perform the PEO process, a homemade external power supply with a maximum power capacity ~20 kW was used under constant voltage conditions (200 AC and 260 DC volts). Primary step coating was carried out in Solution-1 for 15 min and consequently, the samples were treated in Solution-2 with varying concentrations of K_2_ZrF_6_. A flow chart of the whole process is shown in Figure 1. The corresponding coatings prepared in Solution-2 of varying K_2_ZrF_6_ concentration were referred to as K1, K2, K3, K4, respectively. During the course of the PEO process, the cool water flow was maintained to control the constant temperature up to 25 °C. 

### 2.3. Coating Characterization

In order to analyze the effect of K_2_ZrF_6_ on the PEO coatings, the coatings were investigated using various techniques. Phase analysis was carried out using X-ray diffraction (XRD, MiniFlex II, Rigaku, Tokyo, Japan) equipped with a Cu Kα source. Morphology and cross-sections of the samples were examined by scanning electron microscope (Jeol Tokyo, Japan) (SEM), model# JSM-6510. To elucidate the porosity and pore size of the coatings, ImageJ analysis software was used to analyze the surface structure. For micro-hardness measurement of the coatings, 10 different places of each coating cross-section were chosen using a VLPAK2000 Mitutoyo hardness test machine equipped with a Vickers-type indenter under constant loading/unloading rates of 0.025 mN/s with a holding time of 5 s at maximum load. The average micro-hardness value for each coating was obtained from the individual values. In order to investigate the corrosion properties, electrochemical corrosion tests of the PEO-coated specimens were performed by an Electrochemical Testing System(Wonatech, Seoul, Republic of Korea) (1280B) with a conventional three electrode setup; an SCE electrode was used as a reference and a platinum plate as a counter electrode. Coated AZ91 samples, as working electrodes, were immersed in the electrolyte with 3.5 wt.% NaCl. A potentiodynamic polarization test was conducted from 4.0 V below the OCP to 2 V above the OCP at a scan rate of 1 mV/s. The specimens were mounted in the electrochemical cell so that the surface area (0.75 cm^2^) could be in contact with the test solution. Corrosion potential and corrosion current density were obtained through the linear analysis of Tafel approximation using IVman Tafel analysis software (Wonatech, Seoul, Republic of Korea). All the experiments were carried out at room temperature.

## 3. Result and Discussion

### 3.1. Microstructure

**Figure 2:**
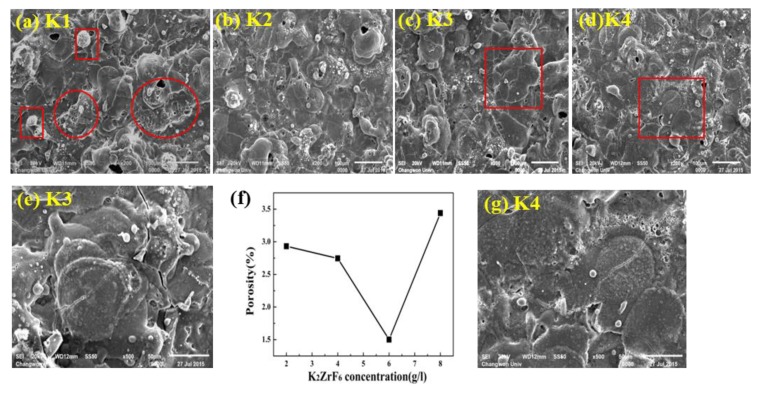
SEM images of the coatings morphologies (**a**) K1, (**b**) K2, (**c**) K3, (**d**) K4. (**e**) Zoom plot of the selected zone of K3 (**f**) Porosity relation with K_2_ZrF_6_ concentration (**g**) Zoom plot of the selected zone of K4.

Surface morphologies of the coatings for varying K_2_ZrF_6_ concentrations (2~8 g/L) are shown in Figure 2a–d. For a lower concentration of K_2_ZrF_6_ (~2 g/L), the surface porosity is higher and the pores exist through the center of the pancakes. The channel routes/pores through the center of pancakes make the pancakes vulnerable to stress and quenching shots. Thus, in most cases, the pancakes are cracked through their centers. It is important to note that the pancake surface is severely damaged by the turbulent eruption of ashes and gases, as shown within the red circles in Figure 2a. In addition, for the K1 sample, the pancakes have no uniform shape but rather, are extended toward the direction of high-pressure flow. Perforated zones formed by the gaseous species eruption can also be observed. In addition to pancakes, small nodules and granular-type structures can also be seen on the surface of K1. Such granular type structures and nodules can be recognized as the ashes formed by the eruptions of the material through discharge channels or by the low-intensity spark-lets. As the concentration increases, the surface porosity decreases together with the decrease in extra-pancake structures. In addition, with increasing K_2_ZrF_6_ concentration, the uniformity of the pancake structure increases. It is important to note that surface zones with perforated morphology caused by the eruption of volatiles and gaseous species were almost completely absent for K3. However, it can be seen that cracks become more stretched for K3, as shown in Figure 2e. Furthermore, pancakes with defined boundaries formed in the case of K3, which suggest a coherent and controlled flow of the viscous material as it erupted from the discharges. Small nodule structures are still present; however, these have a compact and solid structure compared to that for lower concentrations. Upon further increasing the concentration up to 8 g/L, the porosity increases due to the increase in the number of pores; however, the size of the pores decreases for K4. In addition to the cracks and dendrite, well-shaped small crystallite structures and a network of pores appear surrounding each pancake and can also be seen on the K4 surface, as shown in Figure 2g. The surrounding pore-lets appear due to the gas eruption as the pancakes centers are nearly blocked in K4. Thus, it can be noted that a higher concentration of K_2_ZrF_6_ could cause undesired surface structures to form during the PEO process. 

**Figure 3:**
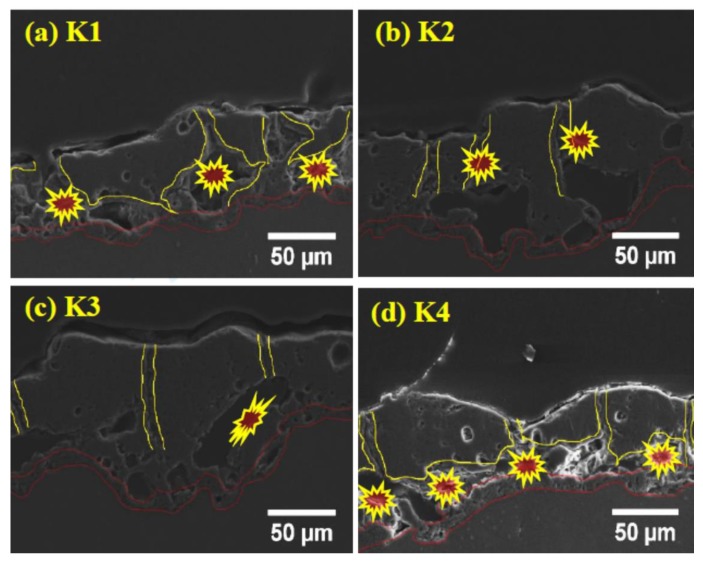
Cross-section of PEO coatings (**a**) K1, (**b**) K2, (**c**) K3, (**d**) K4.

Figure 3a–d shows the cross-section of the coatings for various concentrations of K_2_ZrF_6_. It can be seen that the interface between the coatings and the substrate has a curved boundary for all coatings, which is due to the different rate of alloy dissolution at different points of the substrate material as a result of dissolution of the substrate in the early stages of the process [27]. The barrier layer of all the coatings, irrespective of the K_2_ZrF_6_ concentration, have a similar thickness, as underlined. Large pores/cavities, which are partially connected, can be seen between the inner and outer layers of the coatings (primary and secondary layer). The primary layer is formed as a result of the process in Bath-1, while the secondary coating is formed in post-treatment in Bath-2, as also explained in Figure 1. It was noticed elsewhere that coatings with similar cross-sectional structure, however, with large pores between the inner and outer layers could be observed, when applying only DC or unipolar pulsed DC current modes [28,29]. For a lower concentration up to 4 g/L, the pores and cavities through the cross-sections of the coatings are very large; however, for 6 g/L, the pores size was decreased. A further increase in the concentration caused a decrease in the coating thickness, as well as inner layer damage.

It can be seen from the cross-section observation that for K1 and K2, the shapes of the internal pores and damage are the same; however, with an increase in the concentration, the shapes of the inner layer pores change. It is believed that for lower concentrations, the instability in the discharges gives rise to internal cavities, as the K_2_ZrF_6_ move inside the channel pores. However, with a further increase in the concentration, stable discharges are formed with an increased length and settle on the top surface, deriving anodic material and thus, causing an increase in thickness. Further increasing the concentration of K_2_ZrF_6_ above 6 g/L causes severe damage to the inner and barrier layers due to an increase in acidity caused by the ZrF_6_ ions. The following phenomena are suggested to cause the inner layer damage at the lower concentration and much higher concentration of K_2_ZrF_6_.

- Due to the finite solubility of K_2_ZrF_6_ at higher pH, a colloidal solution is formed, such dielectric particles of K_2_ZrF_6_ obstruct the path of the discharge channels.
- Due to the obstruction and presence of these particles in the discharge effective zones instead of long-time stable discharges, packets of intensive sparks occur randomly due to the breakdown of K_2_ZrF_6_-dominated local zones.
- Increasing the concentration could cause partial dissociation of the K_2_ZrF_6_ due to functionalization and electrophoretic processes, thus allowing the ZrF_6_^2-^ ion to play a part in the conductive transport of electrolyte constituents to the discharge channels and react with the cations thus, offering a minimum resistance and causing stable discharges.

**Figure 4:**
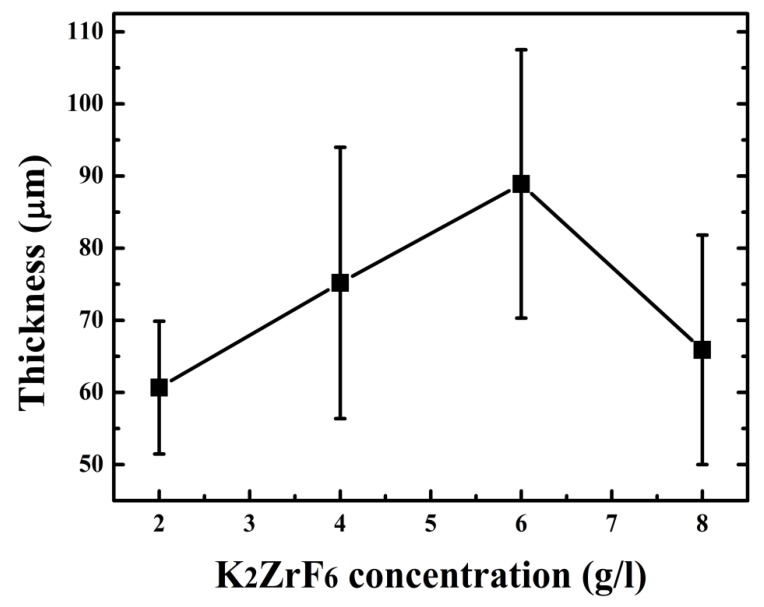
Coating thickness with varying K_2_ZrF_6_ concentration.

Keeping this in view, the concentration was not increased for further experiments. The thickness of the coatings can also be observed from Figure 4. It can be seen that the thickness increases with concentration up to 6 g/L; however, the thickness sharply declined for 8 g/L due to the inner layer damage and thus waste of coating material. Optimized conditions such as an appropriate concentration of the additives assist the uniform increase in thickness, as seen for 6 g/L, which is ultimately responsible for the high protection properties of the coatings.

### 3.2. Phase Analysis

**Figure 5:**
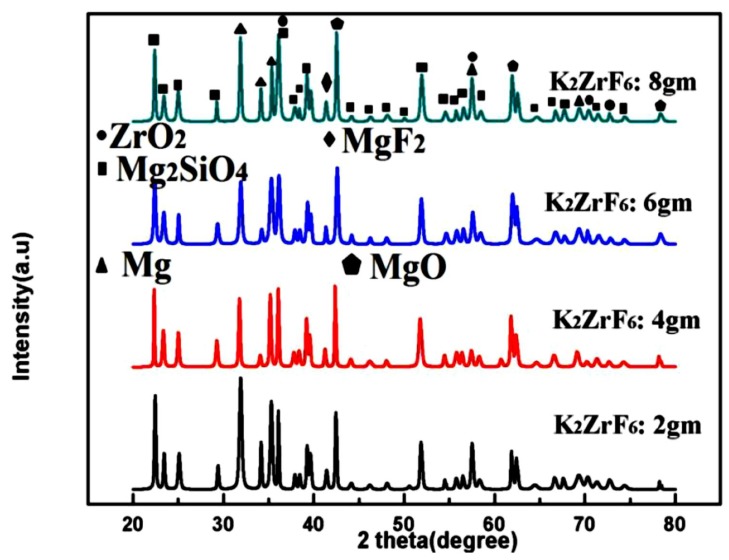
XRD patterns of PEO coatings with varying K_2_ZrF_6_ concentration

Figure 5 shows the XRD patterns of the coated samples. Peaks of Mg_2_SiO_4_, MgF_2_, ZrO_2_ and MgO were identified through matching with reference profiles. It can be seen that the intensity of the Mg peaks greatly decreased for K3; however, it shoots up again for K4 due to highly anodic dissolution of the Mg substrate. In contrast, the intensity of the Mg_2_SiO_4_ peaks was enhanced with increasing concentration. The strongest peak of the Mg_2_SiO_4_ was identified for K4; however, with a simultaneous increase in Mg peak intensities. Thus, it is believed that at a higher concentration of K_2_ZrF_6_, the environment is suitable for reaction between Mg and SiO_2_; however, due to the intensive sparks, outstanding damage to the inner layer caused sufficient rupture of the anodic material from the substrate. Thus, to have a balance between stronger Mg_2_SiO_4_ peaks and a non-damaged inner layer, the concentration of K_2_ZrF_6_ must be limited to 6 g/L. As can be seen, the K3 coating has an intense Mg_2_SiO_4_ peak at 37°; however, with low intensity Mg peaks near 31.9° compared to K1 and K4 coatings where Mg peaks have significant intensity.

### 3.3. Hardness

**Figure 6:**
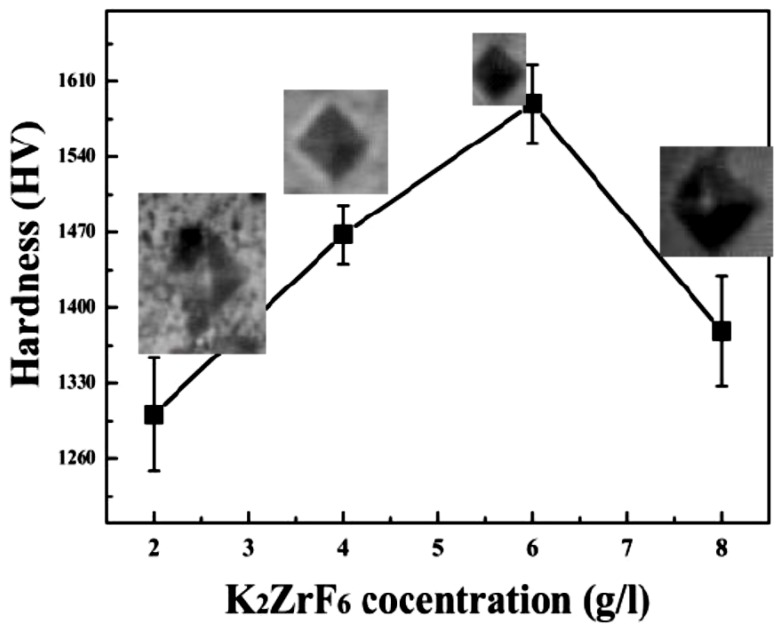
Micro-hardness of PEO coating layers formed in different processes.

Figure 6 shows the average micro-hardness values of the coatings along with the corresponding indentation images. It clearly shows that micro-hardness increases with an increase in concertation up to 6 g/L; however, further increase in the concentration caused a decrease in the micro-hardness values. Furthermore, it can be noted that micro-hardness of the secondary coating is far superior to the primary coatings [30]. Generally, micro-hardness of the secondary coatings was found to be much better than the primary coatings due to the localized nature of the secondary coating that gives rise to intensive sparks and as a result, highly crystalline pancakes. The high micro-hardness value ~1589.45HV, as can be seen from the graph, was obtained for 6 g/L, which can be rarely achieved through other methods or using other PEO electrolyte solutions. As discussed earlier, unlike primary coating, where infinite routes are available for the current, during the secondary process, only limited routes are available for the current Figure 5, which shows the XRD patterns of the coated samples. Peaks of Mg_2_SiO_4_, MgF_2_, ZrO_2_ and MgO were identified through matching with reference profiles. It can be seen that the intensity of the Mg peaks highly decreased for K3; however, it shoots up again for K4 due to highly anodic dissolution of the Mg substrate. In contrast, the intensity of the Mg_2_SiO_4_ peaks enhanced with increasing concentration. The highest peak of the Mg_2_SiO_4_ was identified for K4; however, with a simultaneous increase in Mg peak intensities. Thus, it is believed that at a higher concentration of K_2_ZrF_6_, the environment is suitable for reaction between Mg and SiO_2_; however, due to the intensive sparks, outstanding damage to the inner layer caused sufficient rupture of anodic material from the substrate. Thus, to have a balance between stronger Mg_2_SiO_4_ peaks and a non-damaged inner layer, the concentration of K_2_ZrF_6_ must be limited to 6 g/L. As can be seen, the K3 coating has an intense Mg_2_SiO_4_ peak at 37°; however, with weaker Mg peaks near 31.9° compared to the K1 and K4 coatings, where the Mg peaks have significant intensity.

### 3.4. Corrosion

**Figure 7:**
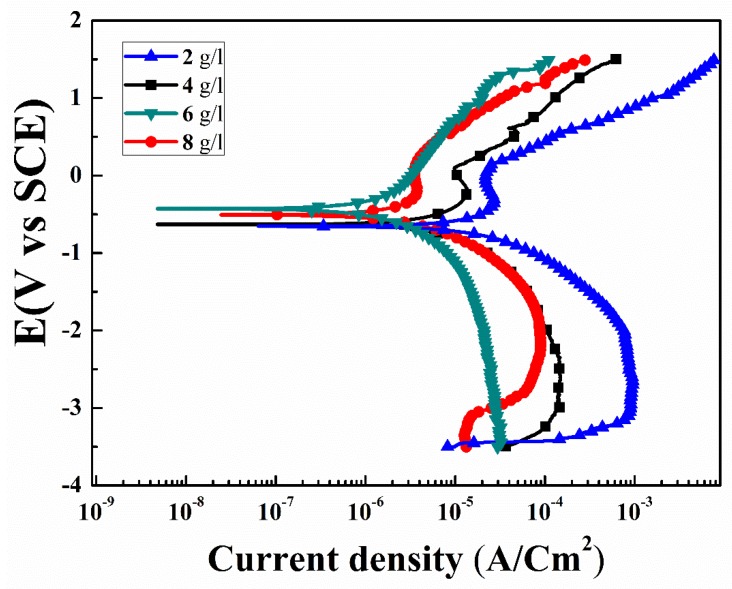
Potentiodynamic polarization curves of PEO coatings with varying K_2_ZrF_6_ concentration.

Figure 7 shows potentiodynamic polarization curves of the specimens for various concentrations of the K_2_ZrF_6_ in 3.5 wt% NaCl solution. The corrosion potential (E_corr_), corrosion current density (I_corr_), and anodic/cathodic Tafel constant (βa and βc) were derived using IVMAN 1.3 Tafel analysis software. All the obtained corrosion parameters are given in Table 1. In general, the high corrosion potential and/or low corrosion current density indicates superior anti-corrosion properties. The anodic curve consisted of various voltage regions associated to its opposition level against the corrosion reactions. The regions comprised are; active, transition and partially trans-passive regions. Similarly, the cathodic part can be divided into a hydrogen evolution and oxygen reduction part. It can be noted that K1, K2 and K4 have the same electrochemical transport trends, as can be seen from the various regions in the anodic curves, i.e., the active region, passivation transition region and partially trans-passive region. However, in contrast, the electrochemical behavior of K3 is quite different, the active region is very limited and there is no specific transition region, rather a continuous shift from the active region to trans-passive region can be observed. The slope of the trans-passive region of K3, as well as the active region, is higher than the relevant regions of the other samples, suggesting efficient and smooth resistance to the corrosion reactions.

Form Figure 7, the passive regions for K1, K2, and K4 (squared) are in the ranges from −0.19 V to 0.12 V, −0.15 V to 0.55 V, −0.30 V to 0.13 V, respectively. These passivation windows are very narrow and cannot offer long-term protection stability against corrosion. It is believed that such narrow windows, possibly could be appear due to the formation of MgCl_2_ or MgH_2_ formation and thus its blockade of the corrosive solution paths. However, due to the large size of the pores in the coating, it could not resist further corrosion processes long-term. To compare the specimens in detail, the corrosion potential and corrosion current density, i.e., (Ecorr = −1.53 V and −0.96 V) and (I_corr_ = 75.01 × 10^−6^ A/cm^2^ and 28.1 × 10^−8^ A/cm^2^), were recorded for the uncoated AZ91D substrate and primary coatings, as previously reported by the author (Table 1) [30]. Samples with secondary step coatings were found to have the highest corrosion resistance. The corrosion potential (E_corr_) of K3 was found ~−0.28 V, compare to the primary coatings E_corr_~ −0.96 V. Similarly, significant increase in the corrosion resistance of the K3 was recorded ~386 × 10^3^ Ω cm^2^. This substantial increase in the corrosion resistance of the specimens with an increase in K_2_ZrF_6_ concentration up to 6 g/L suggests its efficient role in the secondary coatings. The efficient role can be attributed to the high compactness and inert phases in the coatings. However, large internal cavities and damage in the inner layers of the K1, K2 and K4 coatings allowed the corrosion solution to initiate corrosion. The highest corrosion resistance for K3 ~386 × 10^3^ Ω cm^2^ in this work was found to be four-times higher than the corrosion resistance reported by Feryar et al. [26].

## 4. Conclusions

Two-step PEO coatings with varying K_2_ZrF_6_ concentrations were formed on AZ91D Magnesium alloy. For concentrations up to 4 g/L, the surface porosity and inner layer damage were increased due to the obstructive role of the dielectric K_2_ZrF_6_ colloidal component in the solution during the PEO process. However, further increase in the concentration caused a dense, compact inner layer structure together with a decrease in porosity. The optimum role of the K_2_ZrF_6_ for 6 g/L could be attributed to the electrophoretic process involvement. The micro-hardness of the coating prepared for 6 g/L of K_2_ZrF_6_ concentration was found to have the highest values ~1589.45 HV and compact surrounding zone along the indentations. Corrosion resistance of the coatings was investigated and found that due to large pores and cavities in the coatings inner and outer layers for lower concentrations and 8 g/L, the obtained Tafel curves were of the same shape, having narrow trans-passive regions. In contrast, the highest value of corrosion resistance was obtained ~386 × 10^3^ Ω cm^2^ for 6 g/L of K_2_ZrF_6_ concentration, with the lowest values of corrosion current ~2.08 × 10^−8^ A/cm^2^. The highest corrosion resistance for 6 g/L samples was found due to the compact, dense inner layer along with inert phases in the coatings. Coated AZ91 alloy with such significant protective properties can be used in the automobile and aerospace industry after further evaluation.

## Figures and Tables

**Table 1:** Tafel analysis parameters of the PEO coatings.

Con (g/l):	β_a_ (mV/d)	β_c_ (mV/d)	E_corr_ (V)	I_corr_ (A/cm^2^)	R_p_ (Ω.cm^2^)	Hardness (HV)
Primary	47.3	67.4	−0.96	28.1 × 10^−8^	42.9	875
2	45.6	46.4	−0.65	94.1 × 10^−8^	10.6 × 10^3^	1300.92
4	48.8	41.7	−0.51	14.8×10^−8^	65.7 × 10^3^	1467.82
6	41.2	57.5	−0.28	2.08×10^−8^	386 × 10^3^	1589.45
8	42.1	42.5	−0.47	9.16×10^−8^	114 × 10^3^	1378.43
